# Live-cell imaging to measure BAX recruitment kinetics to mitochondria during apoptosis

**DOI:** 10.1371/journal.pone.0184434

**Published:** 2017-09-07

**Authors:** Margaret E. Maes, Cassandra L. Schlamp, Robert W. Nickells

**Affiliations:** 1 Department of Ophthalmology and Visual Sciences, School of Medicine and Public Health, University of Wisconsin – Madison, Madison, Wisconsin, United States of America; 2 Cellular and Molecular Pathology Graduate Program, School of Medicine and Public Health, University of Wisconsin – Madison, Madison, Wisconsin, United States of America; Roswell Park Cancer Institute, UNITED STATES

## Abstract

The pro-apoptotic *BCL2* gene family member, BAX, plays a pivotal role in the intrinsic apoptotic pathway. Under cellular stress, BAX recruitment to the mitochondria occurs when activated BAX forms dimers, then oligomers, to initiate mitochondria outer membrane permeabilization (MOMP), a process critical for apoptotic progression. The activation and recruitment of BAX to form oligomers has been studied for two decades using fusion proteins with a fluorescent reporter attached in-frame to the BAX N-terminus. We applied high-speed live cell imaging to monitor the recruitment of BAX fusion proteins in dying cells. Data from time-lapse imaging was validated against the activity of endogenous BAX in cells, and analyzed using sigmoid mathematical functions to obtain detail of the kinetic parameters of the recruitment process at individual mitochondrial foci. BAX fusion proteins behave like endogenous BAX during apoptosis. Kinetic studies show that fusion protein recruitment is also minimally affected in cells lacking endogenous *BAK* or *BAX* genes, but that the kinetics are moderately, but significantly, different with different fluorescent tags in the fusion constructs. In experiments testing BAX recruitment in 3 different cell lines, our results show that regardless of cell type, once activated, BAX recruitment initiates simultaneously within a cell, but exhibits varying rates of recruitment at individual mitochondrial foci. Very early during BAX recruitment, pro-apoptotic molecules are released in the process of MOMP, but different molecules are released at different times and rates relative to the time of BAX recruitment initiation. These results provide a method for BAX kinetic analysis in living cells and yield greater detail of multiple characteristics of BAX-induced MOMP in living cells that were initially observed in cell free studies.

## Introduction

The tripartite *BCL2* gene family works synergistically to control a cell’s commitment to intrinsic apoptosis. As mediators of apoptosis, *BCL2* genes play a major role in human pathogenesis, including cancer, neurodegeneration, and autoimmune disease [1–3]. Comprised of pro-apoptotic, anti-apoptotic, and BH3-only (effector) proteins, a critical participant within the family is the pro-apoptotic member, BAX, which executes the switch to cell death. Cellular stressors activate BH3-only proteins like tBID, BIM and PUMA, which drive a cell toward apoptosis by both activating BAX and by neutralizing anti-apoptotic proteins [4–6]. Anti-apoptotic family members work to prevent commitment to apoptosis by retro-translocating BAX from the mitochondrial outer membrane (MOM) [7]. When a cell can no longer overcome the stressors within, BAX is recruited to the MOM where oligomer formation occurs, facilitating MOM permeabilization (MOMP) and release of apoptotic molecules like cytochrome c and second mitochondria-derived activator of caspases (SMAC) [8–13]. BAX recruitment to the MOM, including BAX oligomer formation, is the critical event initiating MOMP and the downstream activation of proteolytic caspases in the intrinsic apoptotic program [14].

Many contributions have advanced our understanding of the structure and function of BAX. Composed of nine alpha helices, BAX resides predominantly in the cytosol as an inactive monomer, but will translocate to the MOM during apoptosis, where it becomes active [8–11]. BAX activation occurs through interaction with BH3-only proteins, which cause a conformational change, revealing BAX’s own BH3 domain (α2 helix) and the membrane associated domain (α9) for MOM insertion [15]. BH3-BAX interactions occur at the canonical groove, made of the α2, α3 and α4 helices, and trigger the release of the core-latch domain [16]. Availability of the canonical groove in active BAX leads to homodimerization facilitated by the respective BH3 domains, and oligomerization. High molecular weight oligomers were first shown to exist at mitochondria using crosslinking and gel filtration [13, 17]. BH3-in-groove dimer structures have since been identified using crystallography [16] and confirmed with double electron-electron resonance spectroscopy [16, 18]. Additionally, the BH3-in-groove dimer is required for further assembly of the growing BAX oligomer [19].

MOMP is thought to be the result of BAX-mediated toroidal (proteolipidic) pore formation [20–23]. Until recently, a BAX pore had never been visualized at the mitochondria, however using super-resolution microscopy, STED nanoscopy, and cryo-electron microscopy, BAX was shown to form arc- and ring-like structures and change the MOM topography [24–26]. Although evidence for pore formation is convincing, the mechanism for BAX pore assembly remains to be confirmed. It was proposed that the BAX oligomer grew from a small, monomeric pore to a large, supramolecular pore [27]. Recent studies have shown that a BAX dimer [19], originally proposed to be a BAX monomer [28], is responsible for creating a membrane pore large enough for cytochrome c to be released. Temporally, studies have shown that release of apoptotic molecules is associated with the start of BAX recruitment [29], and that these molecules are released simultaneously [30, 31]. Other studies using artificial or model membranes have suggested that the growing BAX-induced pores release molecules of increasing size [32, 33]. Recent supporting evidence for the concept of growing BAX-induced pores showed that a range of diameters or areas of ring-like BAX structures were present within three different systems for analyzing these structures [24–26]. This work characterizes a clear function for the initial recruitment of BAX and supports the concept of growing BAX oligomers, however the purpose for either the continued recruitment of BAX or the continued expansion of the pore size remain unsolved. Importantly, these events have yet to be explored temporally in great detail.

In contrast to previous work exploring BAX function using biochemical approaches, we used a new approach to dissect the dynamic process of BAX recruitment using fusion proteins in live cell imaging, which allowed us to assess this process at distinct mitochondrial foci. Here we validate and outline a method for BAX recruitment analysis using fusion proteins. We report that the kinetics of BAX recruitment is conserved across different cell types induced to undergo staurosporine-mediated apoptosis. Temporal assessment of the MOMP as a function of BAX recruitment also revealed that the release of apoptotic factors occurs early during oligomer formation, but pro-apoptotic molecules are released at different times in relation to the initiation of BAX recruitment.

## Materials and methods

### Tissue culture cells and nucleofection

D407 cells (immortalized human retinal pigment epithelial cells) were cultured in DMEM (Cellgro, Mediatech, Inc., Manassas, VA) with 3% FBS (Atlanta Biologicals, Norcross, GA) and 1% Antibiotic/Antimycotic (Cellgro, Mediatech, Inc., Manassas, VA) at 37°C and 5% CO_2_. HeLa cells were cultured in DMEM supplemented with 10% FBS and 1% Antibiotic/Antimycotic. HCT116 cells (human colorectal cancer cells) were cultured in McCoy's 5A medium (Lonza, Basel, Switzerland) supplemented with 10% FBS and 1% Antibiotic/Antimycotic. Each nucleofection reaction was comprised of one million cells and 1μg of each plasmid DNA. Nucleofection was performed using the Amaxa Nucleofector (Lonza). D407, HeLa and HCT116 cells were induced for apoptosis using 1 μM staurosporine (STS, Sigma, St. Louis, MO) prepared in DMSO.

D407 cells were a gift from Dr. Aparna Lakkaraju, originating from Dr. R. Hunt at University of South Carolina. HCT116^*BAX-/-/BAK-/-*^ and HeLa wild type cells were a gift from Dr. Richard Youle [34]. All cells used in these studies were wild type for endogenous BAX and BAK proteins, unless otherwise stated.

### Plasmid construction

A construct containing GFP fused in frame with murine BAX was previously described [35]. To generate mCherry-BAX, the murine BAX cDNA was amplified from the GFP-BAX plasmid using primers *5'-GAC TGC GAT GAA GCT TTA ATG GAC GGG TCC GGG* (forward) and 5'-TGC AAG TGC GAA TTC TCA GCC CAT CTT CTT (reverse) with restriction sites *HindIII* and *EcoRI* for ligation into pmCherry-C1 (Clontech, Mountain View, CA). The resulting plasmid (mCherry-BAX) was used to generate a transmembrane BAX mutant (P168A mCherry-BAX) using the QuikChange II site-directed mutagenesis protocol (Agilent Technologies, Santa Clara, CA) to mutate proline 168 to alanine (designated as the α9 mutant). Similarly, amino acids 113–116 in the BAX α5 region were mutated to alanines for the mCherry-BAX α5 mutant and the mCherry-BAX α5/α9 double mutant. Mito-BFP was constructed using an 87 base pair mitochondrial targeting sequence of cytochrome c oxidase subunit VIII (Clontech pDsRed2-Mito sequence) amplified from human cDNA (HCT116 cells) using primers 5’-GAC TGC GAT GAA TTC ATG TCC GTC CTG (forward) and *5*’-TGC TCG TAT TGG ATC CTT CAA CGA ATG GAT C (reverse). Restriction sites *EcoRI* and *BamHI* were used to ligate the mitochondrial targeting fragment into TagBFP-N plasmid (Evrogen, Moscow, Russia). Cytochrome c-GFP (Addgene, plasmid #41182) and SMAC-GFP (Addgene plasmid #40881) were a gift from Douglas Green’s lab [36, 37]. The AIF transcript was amplified from pEntr-AIF (Addgene plasmid # 16182), a gift from Huda Zoghbi [38]. Primers 5’- GAT CGA CGA GAG CTC ATG TTC CGG TGT GGA GGC (forward) and 5’- GGA CAG GTG ACC GGT AAG TCT TCA TGA ATG (reverse) with restriction sites *SacI* and *AgeI* were used to construct AIF-GFP. All constructed plasmids were confirmed through sequencing. See Appendix II for plasmid maps and construction details.

### Immunofluorescence and live-cell staining

D407 cells were fixed using 4% paraformaldehyde in phosphate buffered saline (PBS) for 8 minutes at room temperature, washed three times with 1X PBS then blocked in 10% horse serum (Atlanta Biologicals), 0.3% Triton X-100 in PBS for 1 hour at room temperature. Cells were again washed three times with 1X PBS and incubated in PBS with 0.1% Triton X-100 and 5% horse serum with 1:200 BAX 6A7 mouse monoclonal antibody (Ab-4) (Thermo Scientific, Freemont, CA) overnight at 4°C. After washing, cells were incubated in goat anti-mouse Texas Red secondary antibody (Jackson Immunoresearch, Westgrove, PA) for 2 hours at room temperature. Slides were mounted with Immu-mount (Thermo Scientific).

D407 cells were stained with 50 nM Mitotracker Red FM (Life Technologies, Madison, WI) for 30 minutes at 37°C, washed with 1X PBS, and imaged immediately in recording media (see below) for co-localization with mito-BFP.

### Image acquisition, preparation and analysis

Live-cell and static imaging was performed using the Andor Revolution XD spinning disk confocal microscopy system (Andor, Belfast, Northern Ireland) comprised of the Nikon Eclipse Ti inverted microscope, Nikon objectives, the iXon x3 897 EM-CCD camera, a Yokogawa CSU-X1 confocal spinning disk head, the Andor Laser Combiner with four solid state lasers, an ASI motorized stage with Piezo-Z, and an Okolab CO_2_ cage incubator for temperature and CO_2_ control at 37°C and 5% CO_2_. Cells were plated at a density of 100,000–300,000 cells per well on 4-well chamber slides containing #1.5 optical grade plastic (Ibidi, Madison, WI). Live-cell treatments were staggered for an appropriate time for the cell type to ensure the ability to image each well on a slide. Prior to imaging, phenol-containing media was removed from the well, and replaced with a house made ‘recording media’ (HBSS with 1.26 mM calcium chloride, 0.49 mM magnesium chloride, 0.4 mM magnesium sulfate, no phenol red, 4.5 g/L glucose and 10 mM Hepes) supplemented with appropriate serum concentration. Under temperature and CO_2_ control, time-lapse imaging was performed using a 100X oil objective (numerical aperture = 1.49, temperature collar for 23°C to 37°C) by collecting z-stacks consisting of 20–25 optical sections at 0.22μm (Nyquist) taken every minute for one to two hours. All imaging was performed under the same laser intensity, electron-multiplying gain and exposure time. All time-lapse images were subject to the same background subtraction and Gaussian filter within the IMARIS 7.7 software (Bitplane, Concord, MA), where each filter has its own constant algorithm and threshold setting. The spots function was used to identify and track mitochondrial foci within a cell and extract the maximum fluorescence of the desired fluorophore from each surface. In this study, we define mitochondrial foci as discrete regions where BAX accumulates, which may or may not reflect regions of mitochondrial fission. A subset of mitochondria were selected for computational analysis based on the longevity of the track, whether the track encompassed the recruitment event and remained within the defined space of the cell, and whether the mitochondrial foci were spatially isolated from other mitochondria.

Still images were acquired using either a 60X (Numerical aperture = 1.4) or 100X oil objective, and performed with the same laser intensity, electron multiplying gain and exposure time. All images within a single figure were prepared using the same brightness, contrast and color adjustments in Photoshop (Adobe Systems, Mountain View, CA). To allow for best visualization, cells of interest were cropped from original image files containing multiple cells and placed on a black background.

### Computational analysis

For each mitochondrial dataset, the raw data extracted from the IMARIS program was normalized (to its initial value) to allow for comparison among many mitochondria, then fit to a sigmoid curve. Fitting of the sigmoid curve was done using optimal parameters (*a*, *b*, *k*, *x*_0_, *y*_0_) for a modified sigmoid equation, which allowed inclusion of as many mitochondrial curves as possible. The modified Eq (1) gave the greatest opportunity to fit the most variation in the data, for example, variation in the height and width of the curve. The SciPy library in the Python programming language uses a linear regression model for fitting a sigmoid curve.

$$\text{y}=\frac{a}{b+e^{-k\left(x-x_{0}\right)}}+y_{0}$$ (1)

Metrics were derived from each sigmoid curve, including BAX recruitment initiation, and BAX recruitment rate. BAX recruitment initiation was defined as the x-value where the slope of the maximum rate of the sigmoid curve intersected the minimum boundary, or what is classically defined as the 5% threshold value. Although this definition technically defines a threshold of 12%, we found this calculated value to best represent the data [39]. The BAX recruitment rate was defined as the maximum rate of the sigmoid curve. A similar analysis was performed on decay curves obtained for fluorescently tagged pro-apoptotic proteins released from mitochondria.

Each metric calculating an x-value correlation to time (minutes) was corrected by adding the time between cell treatment and the start of image acquisition. The rate for each stage of the BAX recruitment process was calculated using the curve fit function in SciPy. Standard deviations were calculated using NumPy and plots were generated using Pyplot in the Matplotlib. Scripts were written to import and normalize raw data, fit sigmoid curves, compute all metrics from each curve, compile metrics for population or per cell analysis, and for creating plots.

### Statistical analysis

The datasets for D407, HCT116 and HeLa cells consisted of three or more separate experiments to obtain > 150 mitochondria for each cell type. Statistical power analysis of a preliminary dataset concluded that 150 mitochondria would provide necessary power (0.8) to calculate statistical differences of a minimum of 12%. For mitochondria population comparisons, BAX recruitment rates were Log_2_ transformed to attain normalized distributions and to rectify potential outliers in the populations. Outliers that remained were defined as rates two standard deviations outside the mean, and were removed since the values skewed the datasets. BAX recruitment initiation datasets showed normal distributions, therefore were not transformed. One-way ANOVA was used to calculate p values due to the large sample size, unless otherwise noted. For testing for differences between cytosolic and mitochondrial localization of fusion proteins, a χ^2^ test was used to compare the distributions of cells in different test groups. For calculated statistics based per cell, the standard deviation was calculated for an individual cell and expressed as a percentage of the mean. These data were used to determine the coefficient of variation, which were statistically tested using the two-tailed covariance test. Statistical comparisons between molecule release datasets (n ≥ 46) were also computed using one-way ANOVA.

## Results

### BAX fusion proteins parallel the spatial and temporal localization of endogenous BAX

**Fig 1:**
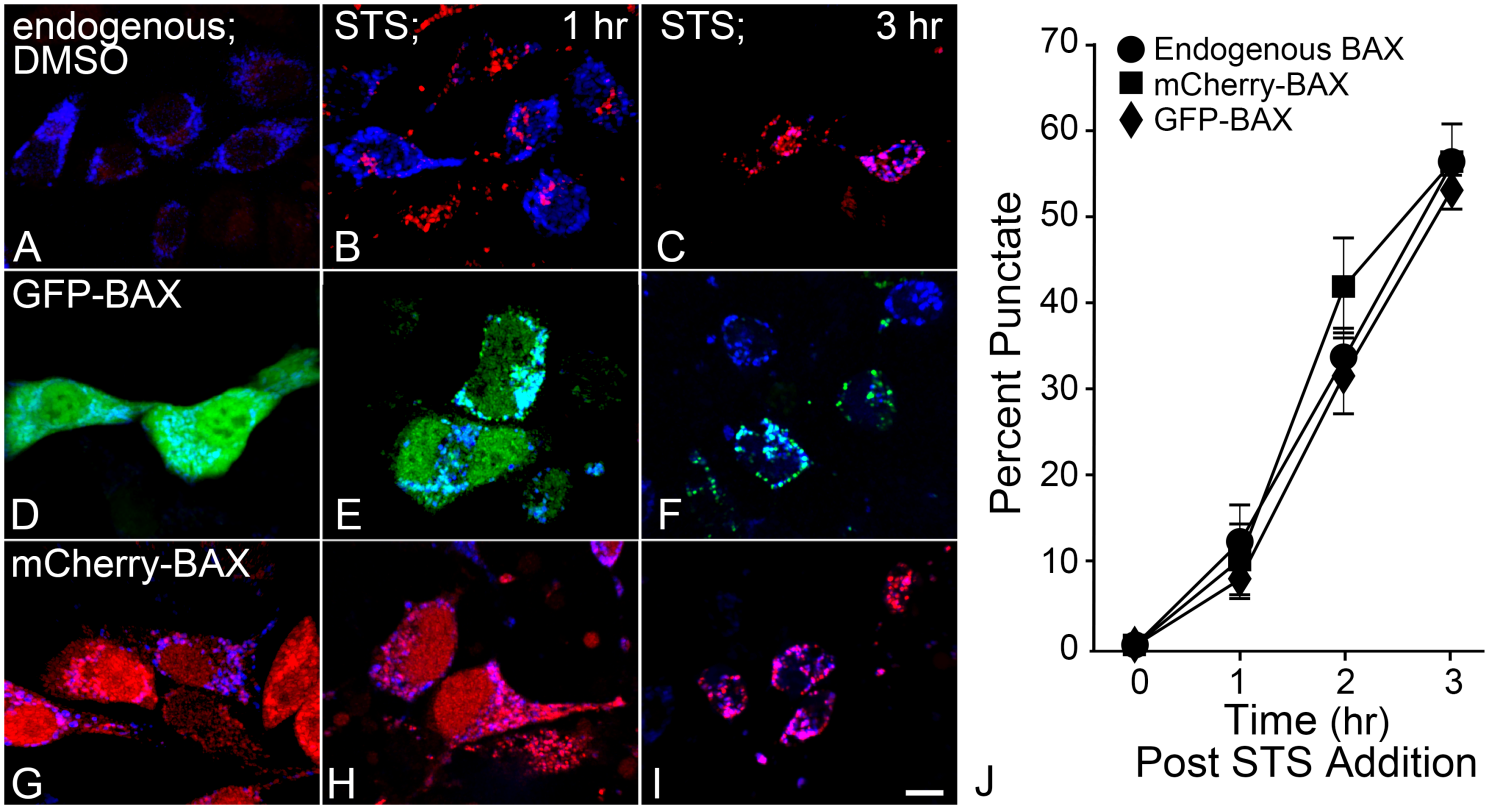
BAX fusion proteins parallel spatial and temporal localization patterns of endogenous BAX. D407 cells were nucleofected with mito-BFP alone or with GFP-BAX or mCherry-BAX. Cells were treated 24 hours later with 1μM staurosporine (STS) for 1, 2, or 3 hours or DMSO control. (A-C) Endogenous activated BAX was monitored using the BAX 6A7 antibody. In DMSO only treated cells (shown after 3 hour incubation) the 6A7 antibody does not cross-react with BAX. Punctate labeling, co-localized to mitochondria, is detected within 1 hour of STS addition. (D-F) Exogenous GFP-BAX and (G-I) mCherry-BAX demonstrate similar localization changes. Size bar = 5 μm. Three independent experiments were quantified. (J) The percentage of punctate cells (number of punctate cells over the total number of nucleofected cells for cells expressing BAX fusion proteins) increased similarly for each time point among all BAX conditions (p > 0.05).

Fusion proteins containing markers such as GFP fused in frame with the BAX N-terminus have been used for nearly 20 years to study BAX function [8, 40], and phenocopy wild type protein function in BAX-deficient cells [35]. To confirm comparable spatiotemporal patterns of BAX fusion proteins and endogenous BAX, activated BAX was monitored after apoptotic induction using the 6A7 antibody. The 6A7 epitope is only exposed after BAX has become active and undergone a conformational change necessary for full recruitment to the MOM [41, 42]. Mitochondria were identified using a plasmid, mito-BFP, which co-localized with Mitotracker Red (S1 Fig). In D407 cells expressing mito-BFP, positive 6A7 staining was detected localized to mitochondria at 1 hour and widespread by 3 hours, after 1 μM STS treatment (Fig 1A–1C). Expressed mCherry-BAX or GFP-BAX under the same conditions showed punctate localization at the mitochondria, comparable to activated endogenous BAX (Fig 1D–1I). The percentages of cells with punctate fusion protein localization were quantified from three separate experiments, and were statistically similar at all time points compared to endogenous BAX recruitment detected with 6A7 immunofluorescence (Fig 1J).

### BAX mutants lacking critical residues impair full BAX recruitment and function

Further evidence that BAX fusion proteins faithfully represented the activity of endogenous BAX protein was assessed using mutants. Two BAX mutant plasmids were constructed. The BAX α9 mutant was created by a substitution of proline 168 for alanine in the 9^th^ α-helix in the C-terminus. In a *BAX*^*-/-*^*/BAK*^*-/-*^ background, this mutation prevents the activation of BAX, normally required for initial MOM interaction, and therefore further BAX recruitment is blocked [43, 44]. In the presence of wild type BAX, however, the α9 mutant BAX can be recruited to the MOM [43]. A second mutant, BAX α5, contained alanine substitutions for leucine-phenyalanine-tyrosine-phenylalanine (LFYF) at amino acids 113–116. These residues were previously shown to be essential for the formation of large molecular weight oligomers consistent with BAX recruitment [45]. The localization and recruitment of each mutant was confirmed in experiments using HCT116 cells lacking both *BAX* and *BAK*. Unlike D407 cells, which show BAX recruitment within 3 hours after the addition of STS, HCT116 cells typically begin to exhibit BAX activation after 9–10 hours of exposure (see below). HCT116^*BAX-/-/BAK-/-*^ cells expressing wild type mCherry-BAX exhibited a punctate pattern 18 hours after STS addition (S2A–S2D Fig), while the BAX α9 mutant (S2E–S2H Fig) and the BAX α5 mutant (S2I–S2L Fig) remained cytosolic.

In separate experiments, we also tested if the BAX mutants could facilitate the release of cytochrome c, which is often considered the critical outcome of BAX activation and recruitment to the MOM [13, 46]. HCT116^*BAX-/-/BAK-/-*^ cells expressing wild type mCherry-BAX and cytochrome c-GFP showed predominantly diffuse localization of cytochrome c-GFP throughout the cell at 18 hours after STS treatment (S3A–S3E Fig), consistent with release of cytochrome c-GFP from the mitochondria. Expression of the BAX α9 mutant and cytochrome c-GFP showed retention of cytochrome c-GFP within the mitochondria accompanied by diffuse BAX α9 at 18 hours (S3F–S3J Fig), indicating a lack of cytochrome c-GFP release. Expression of the BAX α5 mutant and cytochrome c-GFP showed diffuse localization of cytochrome c-GFP within nearly 80% of the cells viewed at 18 hours, however this was not accompanied by detectable mCherry-BAX recruitment such as observed with the wild type protein (S3K–S3O Fig). This outcome may reflect reports that single BAX molecules have the ability to create a pore large enough to release cytochrome c [28] (see Discussion). Because the BAX α9 mutant is unable to integrate into the MOM, to confirm functionality, it was co-expressed with wild type GFP-BAX. Co-expression of the BAX α9 mutant with wild type GFP-BAX restored the ability of the α9 mutant to participate in BAX recruitment (S4A–S4E Fig), as previously reported [43]. This characteristic of the P168A mutant may make using this protein a useful resource to measure BAX recruitment kinetics after the initial activation of WT BAX proteins, and help shed some clarity to previous studies showing that an apparent threshold of BAX was required in a cell to successfully elicit apoptosis [35]. Monitoring this process using time-lapse imaging showed that the BAX α9 mutant did not begin recruiting to the wild type oligomer until wild type BAX had reached a plateau in its own recruitment phase (S5 Fig). The phenomenon of the BAX α9 mutant participating in the BAX oligomer once wild type BAX is present can be abrogated by combining both α5 and α9 mutations into the same protein. The BAX α5/ α9 double mutant did not show detectable BAX recruitment when co-expressed with wild type GFP-BAX (S4F–S4J Fig). Since the α5 mutation blocks BAX from participating in oligomeric growth, the delayed recruitment of the single P168A mutant may reflect a process of oligomeric growth that does not require membrane association through the α9 helix. More studies are required to investigate this possibility. The mutation studies documented here, however, indicate that the BAX fusion proteins and endogenous BAX function similarly. Thus, live cell imaging of fusion proteins can be used to inform the natural process of BAX activation and recruitment during apoptosis.

### Quantitation of BAX recruitment metrics at distinct mitochondrial foci

**Fig 2:**
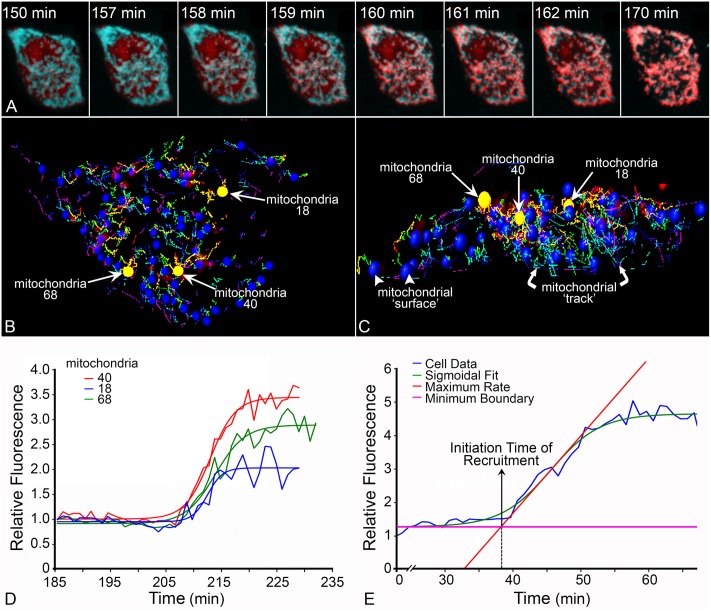
Quantitation of BAX recruitment. (A) Still images from a time-lapse video show a D407 cell expressing mCherry-BAX and a mitochondrial targeted protein, mito-BFP (pseudo-colored teal). The time stamp indicates minutes after the addition of 1 μM staurosporine to induce apoptosis. (B, C) Image analysis software (IMARIS) allows for three-dimensional visualization of identified ‘foci’ within a cell (blue spheres = mitochondrial foci) in the XY (B) or the Z plane (C). The movements of mitochondria are tracked through the Z-stack over time (direction is indicated by red and green lines). (D) A graph of the three mitochondrial foci highlighted in yellow in (B) and (C), demonstrates the accumulation of fluorescent BAX protein at the foci defined by the mito-BFP marker. Raw data is fitted with a sigmoid function. (E) From the sigmoid curve, principal metrics used for analysis can be calculated, including the time of BAX recruitment initiation, the rate of BAX recruitment, and time of BAX recruitment completion.

Time-lapse videos in cells undergoing apoptosis allowed for visualization of the dynamic process from diffuse BAX localization to complete recruitment and localization at the mitochondria. Representative images from a corresponding video show the progression of STS induced apoptosis in a D407 cell nucleofected with mCherry-BAX and mito-BFP (S1 Video, Fig 2A). For quantitation, IMARIS 7.7 image analysis software was used to identify mitochondrial foci (mito-BFP) within a cell and track them in the three dimensional Z-stack throughout the duration of the time-lapse video (Fig 2B and 2C). From each of the mitochondrial foci, GFP- or mCherry-BAX, fluorescence intensity was collected for all time points, normalized and fitted with a sigmoid curve (Fig 2D). Important parameters for analyses were defined from the sigmoid curve, including the time of initiation and rate of BAX recruitment (Fig 2E) (see Methods). It is important to note that the dynamics of BAX recruitment visualized in these experiments may reflect an equilibrium of BAX actually being recruited against the rate of BAX retrotranslocation. Retrotranslocation is a phenomenon describing removal of BAX from the MOM that has been documented in healthy cells and is controlled by anti-apoptotic proteins such as BCL-X [7]. Once apoptosis is initiated and the anti-apoptotic proteins are sequestered by activated BH3-only proteins, the rate of retrotranslocation is presumably reduced to a negligible value.

**Fig 3:**
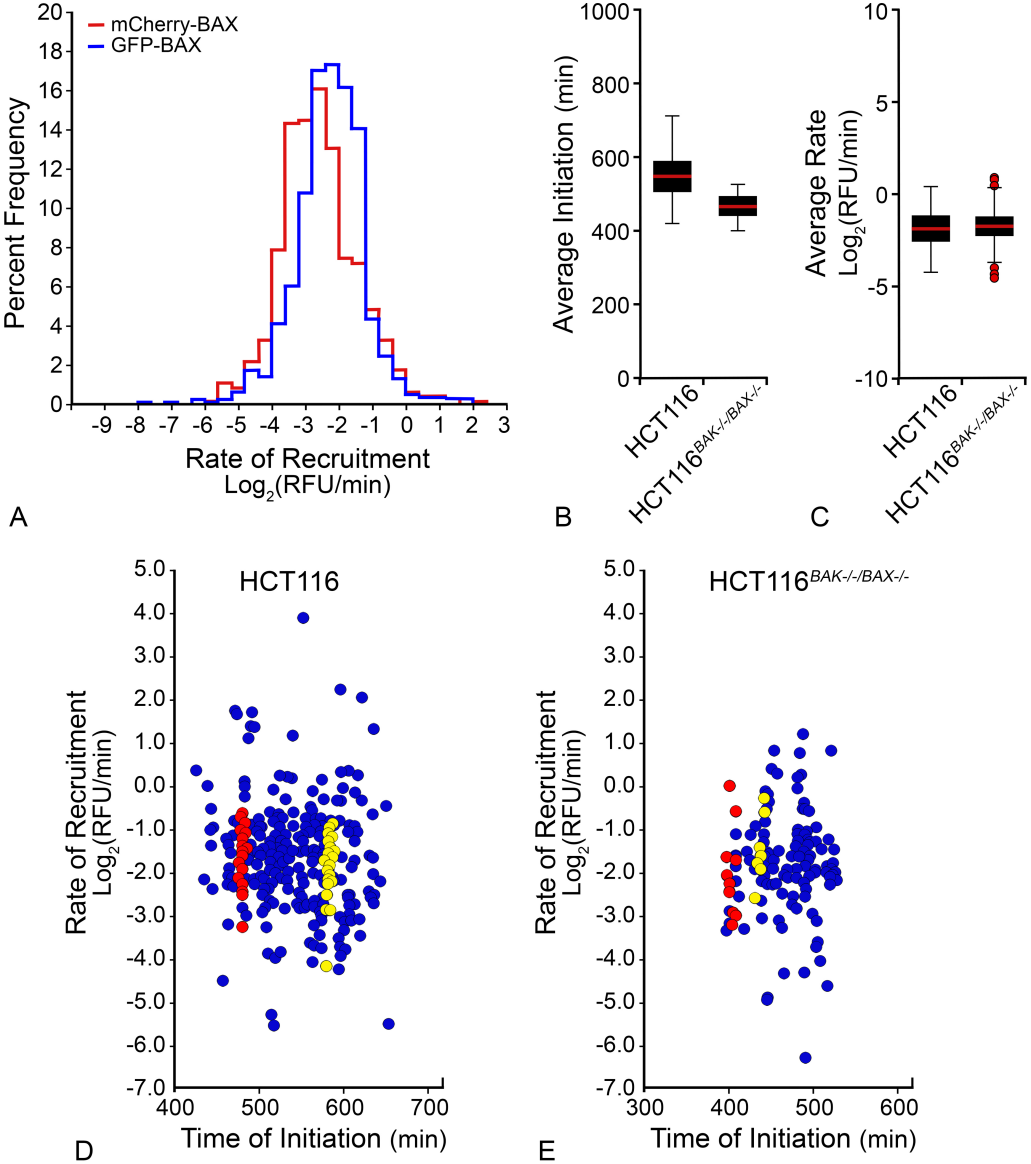
Effects of fluorescent tags and endogenous protein on BAX recruitment kinetics. (A) To determine the effect of fluorescent tags on BAX recruitment rates, the rate of BAX recruitment was calculated for two populations of mitochondria from D407 cells, expressing either mCherry-BAX (red curve) or GFP-BAX (blue curve). A graph depicts the outline of the frequency plot for each population of mitochondria (n = 680 and 727 mitochondria for GFP-BAX or mCherry-BAX, respectively). GFP-BAX recruitment exhibited a statistically faster rate of recruitment than mCherry-BAX (p < 0.05). (B-E) To determine the effect of endogenous BAX protein on GFP-BAX recruitment rates, two populations of mitochondria were compared from wild type HCT116 and HCT116^*BAX-/-/BAK-/-*^ cells. Box and whisker plots compare the (B) average initiation time for each population (p < 0.001) and (C) the average rate of BAX recruitment. There was no difference in recruitment rates between wild type HCT116 and HCT116^*BAX-/-/BAK-/-*^ cells. The average rates and initiation times were derived from the population of mitochondria shown in (D and E). Each individual marker represents one mitochondrial focus. Red and yellow markers identify two separate cells from each population. Data from foci originating from the same cell are vertically stacked indicating nearly identical times for the initiation of BAX recruitment within a cell, but that recruitment occurs at different rates at individual foci (n = 424 and 132 mitochondrial foci analyzed for HCT116 and HCT116^*BAX-/-/BAK-/-*^ cells, respectively).

To determine if individual fluorescent tags affected the recruitment of the fusion proteins, kinetics were evaluated from two populations of mitochondria from D407 cells undergoing STS-induced apoptosis, expressing either mCherry-BAX or GFP-BAX. While similar, GFP-BAX exhibited a significantly faster average rate of recruitment (-1.87 ± 1.0 Log_2_(RFU/min)) than the mCherry-BAX (-2.2 ± 1.4 Log_2_(RFU/min)) (p < 0.05) (Fig 3A). Consequently, for all subsequent comparative experiments, BAX fusion tags were kept consistent. To address an effect from endogenous BAX or BAK, recruitment kinetics were evaluated from three populations of mitochondria from either wild type HCT116 or HCT116^*BAX-/-/BAK-/-*^ double knock-out cells treated with STS, each expressing exogenous GFP-BAX. The times of initiation for the two populations were statistically different by one-way ANOVA, but all fell within a range between 400–500 minutes after apoptotic induction (Fig 3B, p < 0.001). There was no statistical difference in the rate of GFP-BAX recruitment between HCT116 wild type and HCT116^*BAX-/-/BAK-/-*^ populations (Fig 3C, p = 0.110), indicating that endogenous pro-apoptotic homologs do not affect recruitment of the exogenous BAX fusion protein. The lack of changes between double knock-out and wild type cells provides compelling evidence that endogenous BAX and BAK do not affect the rate of BAX recruitment. In Fig 3D and 3E, the stratification of recruitment rates for each population is shown as a function of time of initiation. Data from the mitochondrial foci analyzed in two individual cells per condition are highlighted and demonstrates that initiation appears synchronous within a given cell while rates are variable. This phenomenon holds true regardless of endogenous BAX or BAK expression and is addressed in greater detail in the following section.

### BAX recruitment kinetics reveal synchronous initiation, but variable rates of recruitment in cells, regardless of cell type

**Fig 4:**
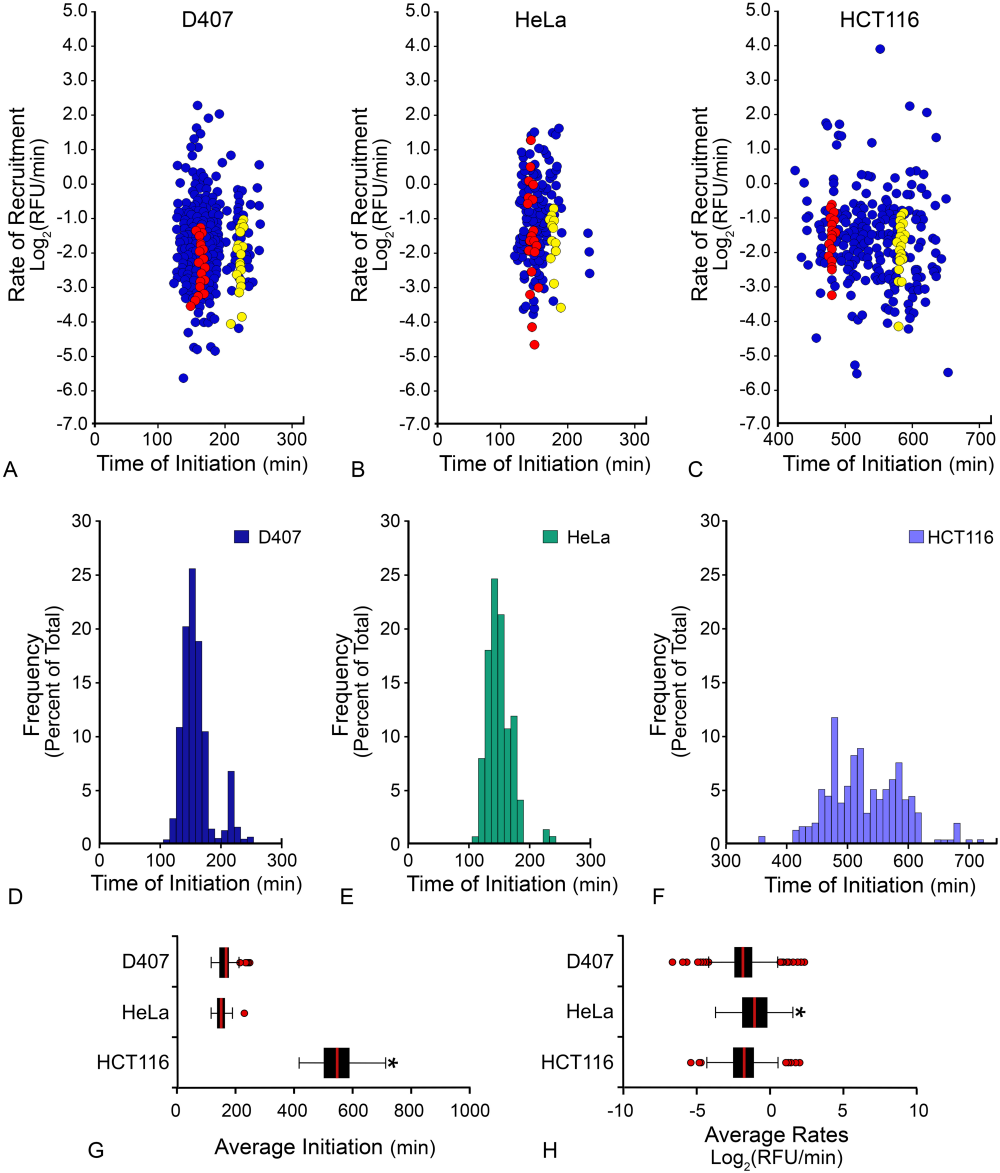
Initiation of BAX recruitment and BAX recruitment rates in three different cell types. (A) GFP-BAX recruitment kinetics were quantified for three different cell types; D407, HeLa, and HCT116. All cells were wild type for endogenous BAX and BAK proteins. Apoptosis was induced by exposure to 1 μM staurosporine (STS). Times given represent the minutes elapsed after STS addition. (A-C) A scatterplot for each cell type shows the time of BAX recruitment initiation on the X-axis and BAX recruitment rate on the Y-axis. Each circular marker represents individual mitochondrial foci from the population (n = 680, 156, 336 mitochondrial foci for D407, HeLa, HCT116, respectively). For each cell type, red and yellow markers show all the mitochondria from two individual cells, demonstrating synchronous recruitment initiation times, but stratified recruitment rates. (D-F) Frequency plots for each cell type (D407, HeLa, HCT116, respectively) show the varied time of BAX recruitment initiation for each population. Each cell type exhibits a distribution of initiation times distinctly different from each other (p < 0.001). (G) A box and whisker plot shows the BAX initiation times for D407, HeLa, and HCT116 cell populations. Each population is significantly different from each other (p < 0.001). (H) A box and whisker plot shows the BAX recruitment rates for D407, HeLa and HCT116 cell populations. HeLa cells had a significantly faster average rate of recruitment compared to either condition (*p < 0.001).

GFP-BAX recruitment kinetics were analyzed in three different cell types. Apoptosis was induced using STS for D407, HeLa and HCT116 cells. All cells used in this study contained endogenous WT BAX and BAK proteins. Scatter plots depict the rate of BAX recruitment as a function of BAX recruitment initiation time for populations of mitochondrial foci analyzed from each of the three different cell populations (Fig 4A–4C, note that Fig 4C is the same data represented in Fig 3D). Varying BAX initiation times and BAX recruitment rates were evident among the three different cell types. Individual cell populations exhibited a range of initiation times as evidenced by rate distribution (Fig 4A–4C) and mitochondria frequency plots (Fig 4D–4F). The difference in average initiation time held true even when apoptosis was induced by the same stimulus (STS treatment of D407 and HCT116 cells, p < 0.001), or when the population distributions overlapped (D407 and HeLa cells, p < 0.001) (Fig 4G). The distribution of recruitment rates at mitochondrial foci, within all cell types, was also highly variable. Nevertheless, D407 (-1.8 ± 1.0 Log_2_(RFU/min)) and HCT116 cells (-1.8 ± 1.2 Log_2_(RFU/min)) yielded statistically similar average rates (p = 0.392). In comparison to D407 and HCT116 cells, HeLa cells (-1.0 ± 1.3 Log_2_(RFU/min)) yielded a significantly faster average rate of BAX recruitment (p < 0.001, Fig 4H).

**Fig 5:**
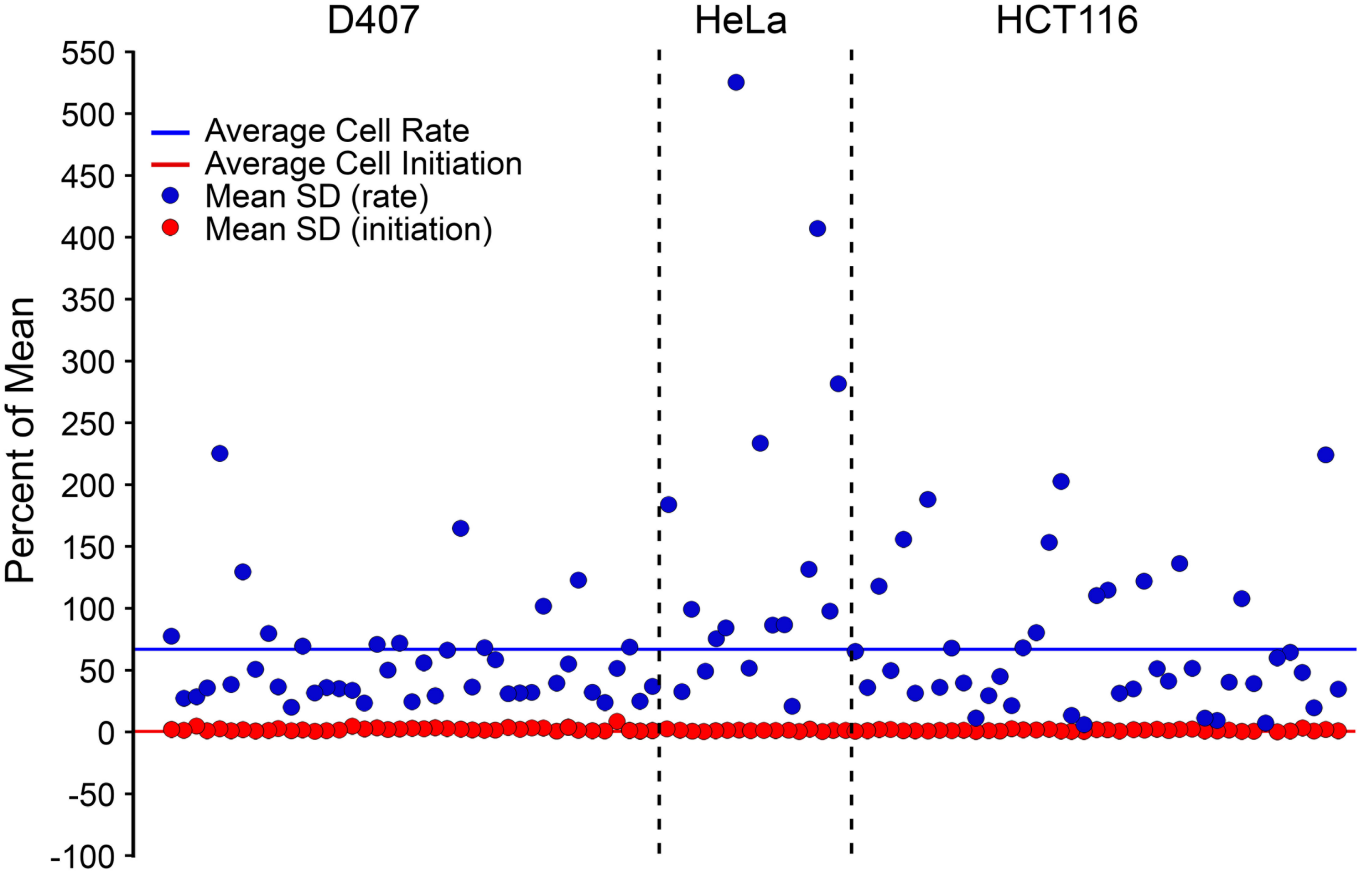
Coefficient of variation analysis. The coefficient of variation describes the variability for each metric within an individual cell (n = 41, 24, 42 cells for D407, HeLa, and HCT116, respectively). This statistic is normalized to a percentage of the means, which allows for direct comparison between different metrics. Each cell is represented by a single red and blue point. The average coefficients of variation of all cells within a cell type for BAX recruitment rates were 57%, 150%, and 67% for D407, HeLa, and HCT116, respectively. The average coefficient of variation of all cells within a cell type for the initiation of BAX recruitment were 2%, 5%, and 1% for D407, HeLa, and HCT116 respectively, with a statistical difference between D407 cells when compared to HCT116 (p < 0.05) and between HeLa cells when compared to any other cell type (p < 0.001). The mean of each metric for all cells is shown as a solid red or blue line. Comparison between the two metrics for all cells, regardless of cell type, showed a statistical difference (p < 0.001) by the two-tailed covariance test. These data indicate that while BAX recruitment is initiated at unique times in different cell types (Fig 4), once activated, recruitment is initiated relatively synchronously within a given cell. The rate of recruitment within the same cell, however, can vary dramatically. These features appear to be conserved across different cell lines.

Evaluation of these data on a per cell basis revealed that the initiation of recruitment was nearly synchronous for all the mitochondrial foci examined within a single cell, but the rates exhibited during recruitment were still highly variable. A representative video highlights the synchronous initiation of GFP-BAX recruitment in each cell type (S2 Video). Graphically, this phenomenon can be inferred from the examples of two individual cells, plotted in red and yellow, from each population (Fig 4A–4C). Statistically, we calculated the coefficient of variation for each metric for each cell in three segregated cell populations, which is represented graphically in Fig 5. The average coefficients of variation for D407, HeLa and HCT116 for BAX recruitment rates were 57%, 150%, and 67%, and for BAX initiation times were 2%, 5%, and 1%, respectively, indicating that there was a high degree of variability among BAX recruitment rates, but little variability of BAX initiation times within cells. Additionally, the intrinsic variability between the two metrics was not the same for any cell type by the two-tailed covariance test (p < 0.001 for D407, HeLa, HCT116). Taken together, these data suggest that irrespective of cell type or the time after stimulus to initiation, BAX recruitment possessed two conserved qualities; BAX recruitment was initiated synchronously within a cell, and BAX recruitment rates were variable within a cell.

### Release of apoptotic molecules occurs at different times during BAX recruitment

**Fig 6:**
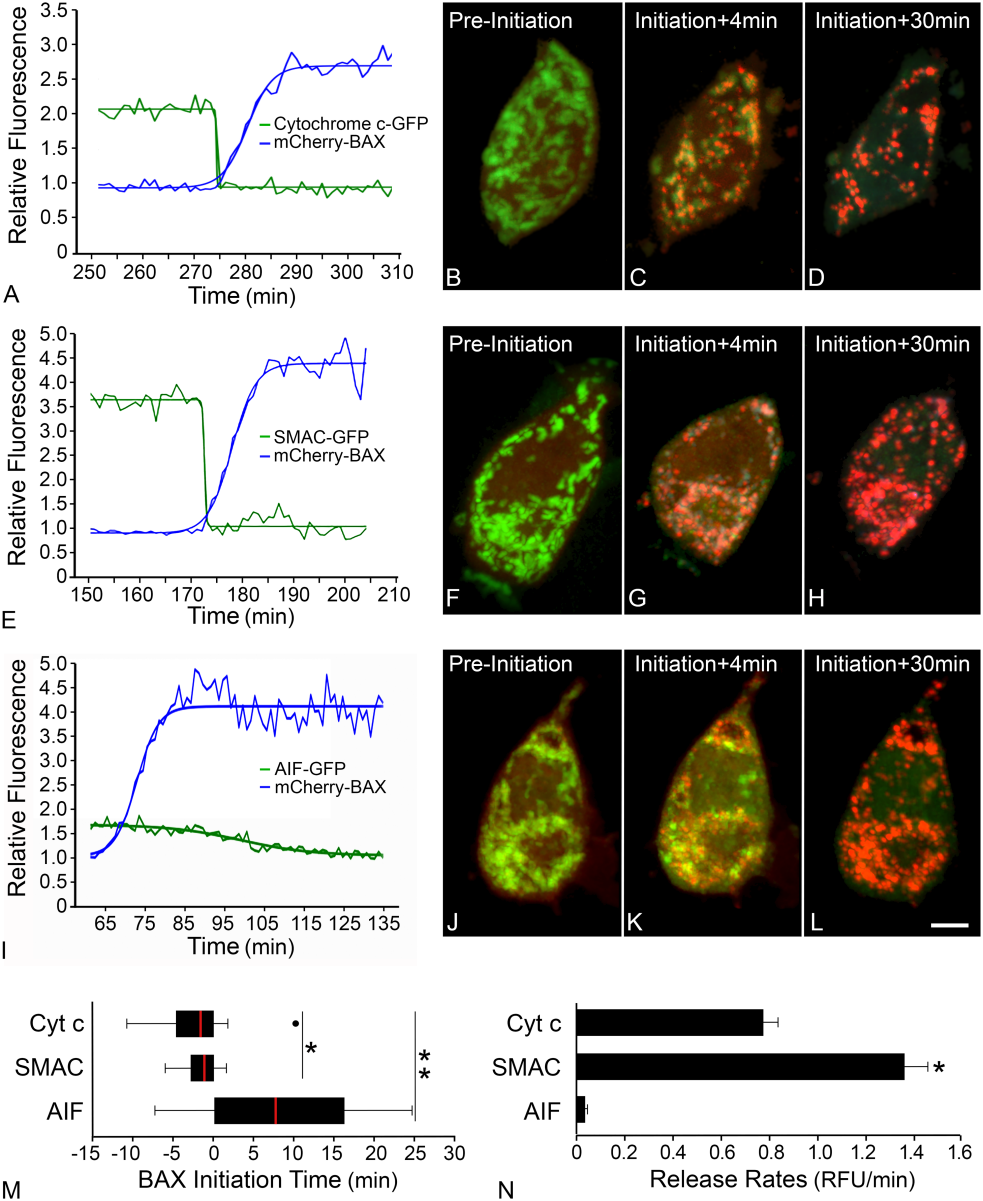
Cytochrome c, SMAC and AIF are released at different times during BAX recruitment. Time-lapse videos were collected from D407 cells expressing mCherry-BAX, mito-BFP and one of three GFP-tagged apoptotic molecules that are released from mitochondria after being challenged with 1 μM staurosporine. Representative graphs indicate the release of apoptotic molecules (A) cytochrome c-GFP, (E) SMAC-GFP, and (I) AIF-GFP, and the recruitment of mCherry-BAX at one mitochondrial focus. (B-D) Representative images show (B) cytochrome c-GFP localized at the mitochondria (pre-initiation), and (C) diffuse cytochrome c-GFP four minutes after BAX recruitment initiation and (D) 30 minutes after BAX recruitment initiation. (F-H) Images from a time-lapse video of SMAC-GFP and mCherry-BAX (F) pre-initiaton, (G) four minutes and (H) 30 minutes after BAX recruitment initiation, show the same localization patterns as cytochrome c-GFP. (J-L) Images from a time-lapse video of AIF-GFP and mCherry-BAX show (J) AIF-GFP localized at the mitochondria (pre-initiation), (K) AIF-GFP partially retained in the mitochondria four minutes after BAX recruitment initiation and (L) diffuse AIF-GFP 30 mins after BAX recruitment initiation. For all three datasets, the time of molecule release was compared to the time of BAX recruitment initiation. (M) A box and whisker plot shows that cytochrome c-GFP and SMAC-GFP are released -2.5 ± 3 mins and -1.3 ± 1.6 mins before mathematical assignment of the initiation of BAX recruitment, respectively, while AIF is released at 10.3 ± 11 mins after recruitment initiation. Each molecule’s relative release time was significantly different from the others by one-way ANOVA (p** < 0.01; n = 53, 52, 46 mitochondria foci for cytochrome c, SMAC, and AIF, respectively). Cytochrome c and SMAC release times were also significantly different (p* < 0.05). (N) A bar graph showing the rate of apoptotic molecule release for each molecule. Cytochrome c was released at a rate of -0.78 ± 0.46 RFU/min, SMAC at -1.36 ± 0.72 RFU/min and AIF at -0.04 ± 0.07 RFU/min (n = 54, 52, 51 mitochondria foci respectively). All were significantly different (p < 0.001), while SMAC exhibited a significantly faster release rate when compared to the other molecules (*p< 0.001).

BAX recruitment kinetics were quantified in tandem with release kinetics of the apoptotic molecules cytochrome c, SMAC, and apoptosis inducing factor (AIF) in D407 cells after STS induced apoptosis. Representative graphs show the overlap of a BAX recruitment curve and a molecule release curve from the same mitochondrial focus within a cell for cytochrome c-GFP (Fig 6A), SMAC-GFP (Fig 6E), and AIF-GFP (Fig 6I). Each graph is paired with representative images from a time-lapse video indicating the time before BAX initiation (pre-initiation), 4 minutes post-initiation, and 30 minutes post-initiation for cytochrome c-GFP release (Fig 6B–6D), SMAC-GFP (Fig 6F–6H), and AIF-GFP (Fig 6J–6L). A time-lapse video demonstrates the temporal relationship between cytochrome c and BAX recruitment (S3 Video). Notably, the average mCherry-BAX recruitment rates were similar in cells with or without co-expression of these GFP-tagged apoptotic molecules (p = 1.0). For individual mitochondrial foci, the time of molecule release was compared to the time of BAX initiation. Analysis of at least 46 mitochondrial foci in each condition showed that cytochrome c and SMAC were released at -2.5 ± 3 minutes and -1.3 ± 1.6 minutes, respectively, before BAX recruitment initiation, while AIF was released at 10.3 ± 11 minutes after BAX recruitment initiation (Fig 6M). Release times between all molecules were significantly different, (p < 0.01) and were staggered according to molecular mass, with the smallest molecule being released the earliest. Additionally, we observed significantly different rates of release for each molecule (Fig 6N, p < 0.001). Cytochrome c was released at a rate of -0.78 ± 0.46 RFU/min, SMAC at -1.36 ± 0.72 RFU/min and AIF at -0.04 ± 0.07 RFU/min. These results demonstrate a difference in release time relative to BAX recruitment initiation, and release rate for each apoptotic molecule.

## Discussion

In this report, we describe the use of high speed live-cell imaging to measure the process of BAX recruitment to the MOM in apoptosing cells. While others have used fluorescently tagged BAX fusion proteins to monitor BAX activation and translocation to the MOM [8], our studies mark an increase in resolution that has allowed for the kinetic analysis of the recruitment process at multiple individual sites in a single cell. We posit that the image analysis of BAX recruitment also reflects the formation of BAX oligomers. This supposition is partly based on the historical evidence that when BAX is active and present at the MOM, BAX dimers form leading to arc- and ring-like structures and eventually large molecular weight homo-oligomers [9, 16, 19, 24]. Also, in our studies, we found that the process of recruitment was inhibited by a mutation that has been shown to prevent oligomerization (the α5 mutant) when expressed in a *BAX/BAK*-deficient background [45].

The use of BAX mutants supports the concept that BAX activation and function is a multi-ordered process. The α9 mutant blocks the initial insertion of activated BAX into the MOM, thus preventing the formation of pores and the release of cytochrome c. This region of BAX is apparently not required for further recruitment of BAX into a growing oligomer, which is supported by evidence that cells can recruit the α9 mutant protein when they also express wild type protein. Live-cell analysis of the α9 mutant in the presence of wild type BAX, further dissected this relationship, demonstrating that the α9 mutant only begins to participate in the recruitment process at the plateau, or completion phase of the wild type BAX protein. This further supports a multi-ordered process of BAX recruitment, where an initial insertion phase is followed by a phase of accumulation at the site of oligomer formation. This phenomenon is eliminated if the α9 mutant also carries the α5 mutation. Conversely, our results show that the α5 mutant, which is unable to form into an oligomer, does allow for the release of cytochrome c. Our interpretation is that preservation of the sequence of the C-terminus allows for this protein to interact with the MOM and leads to initial pore formation. Xu and colleagues, [28], used membrane containing nanodiscs designed to accommodate a single BAX molecule and demonstrated that BAX insertion was able to create a pore large enough to allow for the release cytochrome c. Thus the α5 mutant may have been able to form pores and release cytochrome c without forming puncta commonly associated with oligomer formation. Our observation also seems to contradict a separate study by George and colleagues of the apoptotic ability of the α5 mutant [45]. In that study, apoptosis was evaluated by the formation of pyknotic or fragmented nuclei in DAPI stained cells at a single time point after STS addition. The results showed no significant increase in cell death, contrary to what we would predict if cytochrome c was being released. Lack of pyknotic cells in the George study could be explained by a delay in the downstream apoptotic cascade. If a large molecular weight BAX oligomer has additional roles, such as to promote mitochondrial fragmentation, perhaps the BAX α5 mutant cannot complete this task rapidly and nuclei remain intact for longer. Further experimentation to dissect the exact number or structure of wild type or mutant BAX molecules at each mitochondrial focus analyzed would be incredibly valuable, however it is outside the resolution of the spinning disk confocal microscope.

In our temporal analysis, we also observe the rapid release of pro-apoptotic molecules from mitochondria, early in the BAX recruitment process. Cytochrome c and SMAC were released even prior to our declaration of the initiation of BAX recruitment. We do not interpret this result as meaning that release is BAX independent. Instead, we believe that this early release reflects limits of our analysis to detect individual or small numbers of BAX molecules that have been recruited, and the methods we used to provide statistical confidence that BAX recruitment had achieved a level over the background signal (see Methods). Because inactive monomeric BAX is cycling on and off the membrane [7] and is also fluorescently tagged, the baseline fluorescent readings in our studies always include some number of BAX molecules, however the change in fluorescence cannot be detected until more than baseline is present at the MOM. The change in fluorescence is likely detected after the dimerization process has occurred, and therefore after release of cytochrome c. Previous studies using less sensitive methods also reported that cytochrome c was released prior to BAX redistribution [29, 47]. More importantly, Xu et al. [28] demonstrated that single activated BAX molecules, constrained in lipid nanodiscs, were able to create proteolipidic pores 35 Å in diameter. Additionally, Zhang et al [19] showed that a BAX mutant, T182I, only able to form BAX BH3-in-groove dimers (but not oligomers), was able to release cytochrome c, but not SMAC. Therefore it is likely that a BAX dimer is sufficient to create pores at least 35 Å in diameter, which would release cytochrome c-GFP, supporting our observations of cytochrome c release prior to detectable BAX recruitment.

We also observed that the pro-apoptotic molecules were released at different times relative to the BAX recruitment process. This contradicts previous work reporting simultaneous release of these molecules [30, 31]. There are several possible explanations for this difference. First, we monitored the kinetics of molecule release as a function of BAX recruitment kinetics, thus providing a temporal anchor point for analysis. Second, the data collected in previous studies presented average values for whole cell fluorescence changes, while we specifically monitored changes at individual mitochondrial foci. Third, the best temporal resolution previously recorded for molecule release was from a single Z-plane, taken every three minutes [30]. We recorded every 60 seconds from an entire Z-stack. This increase in recording speed, added to capacity to collect data from a three dimensional space, could explain how we were able to observe a difference in the release times, particularly between cytochrome c and SMAC, which appear to be released at least 1 minute apart from each other. We did not, however, directly measure the release of both molecules from the same cell. This kind of measurement would require using different fluorescent tags, which may add a confounding variable to the kinetic analysis such as the subtle difference in recruitment rates we detected between mCherry-BAX and GFP-BAX. Nevertheless, such an analysis would be warranted to confirm the temporal differences that we are reporting relative to the BAX recruitment process.

Interestingly, in relation to the initiation of BAX recruitment, the order of cytochrome c (12 kDa), SMAC (21 kDa), and AIF (54 kDa) release followed a sequence from lowest to highest molecular weight. Evidence using artificial or isolated membrane systems indicates that the growing BAX oligomer is associated with increasing pore size, leading to the release of sequentially larger tracer molecules [32, 33]. Assuming that the GFP tag (30 Å diameter) has no impact on release kinetics through the minimal sized BAX pore (35 Å diameter), we could interpret a similar condition is also evident in living cells. While this may hold true for globular proteins like cytochrome c and AIF, SMAC is a long cylinder and actually has the shortest axis (~20 Å) of all three molecules examined. It is possible that SMAC is released predominantly as a homodimer, which would be consistent with the requirement for a pore with increasing diameter. At this point we cannot rule out that other factors may be affecting the release of molecules of different size and shape, including where they initially reside in the mitochondria (i.e., in the matrix or intermembrane space). Our data do indicate, however, that the dynamics of the MOMP change as a function of increasing BAX recruitment.

Collectively, our results showed that the kinetics of BAX recruitment can be monitored in a system using live cell imaging of BAX fusion proteins and that this process proceeds synchronously, but with variable rates, regardless of cell type. Importantly, this analysis has provided validation of many of the characteristics of BAX obtained from cell free systems as also occurring in living cells.

## Supporting information

S1 Fig Mito-BFP co-localized with Mitotracker. Mito-BFP encodes a transcript containing the mitochondrial targeting sequence for cytochrome c oxidase subunit VIII fused in frame to BFP. Mitotracker Red FM is a live-cell imaging dye that stains the mitochondria. A representative image of a D407 cell expressing (A) Mito-BFP and also stained with (B) Mitotracker Red, shows colocalization of the two markers in (C) a merged image. Size bar = 3 μm.
(TIF)

S2 Fig BAX mutants fail to recruit to MOM after staurosporine treatment in HCT116^*BAX-/-/BAK-/-*^ cells. HCT116^*BAX-/-/BAK-/-*^ cells were nucleofected with different BAX mutants to provide evidence for functioning BAX fusion proteins. Images were taken 18 hours post addition of 1 μM staurosporine (STS), at a time when a majority of these cells exhibit BAX activation, and presented as three panels, a merged image along with individual channels. (A-C) Wild type (WT) mCherry-BAX showed recruitment to the MOM indicated by the punctate pattern co-localized with the mitochondria. (D) A pie chart showing the distribution of the cells showing either predominantly cytosolic (C) or predominantly mitochondrial (M) localization. (E-G) The BAX α9 mutant failed to fully recruit to the MOM, indicated by diffuse localization of BAX α9. (H) Pie chart of scored cells. (I-K) The BAX α5 mutant also failed to recruit to the MOM, indicated by diffuse localization of BAX α5. (L) Pie chart of scored cells. Both mutants show a significantly different localization pattern of BAX compared to the WT protein under these conditions (χ^2^ test, p < 0.0005). Size bar = 5 μm.
(TIF)

S3 Fig Cytochrome c-GFP localization in the presence of BAX mutants after staurosporine treatment in HCT116^*BAX-/-/BAK-/-*^ cells. HCT116^*BAX-/-/BAK-/-*^ cells expressing wild type or mutant mCherry-BAX, cytochrome c-GFP and mito-BFP were challenged with 1μM staurosporine (STS) and observed at 18 hours after treatment. In healthy cells, the cytochrome c fusion protein is localized to mitochondria (see Fig 6 and S3 Video). (A-D) Wild type mCherry-BAX exhibits punctate BAX and diffuse cytochrome c-GFP labeling. The merged image (A) is followed by separate channels. (E) A pie chart showing the scoring of cells exhibiting predominantly cytosolic distribution of cytochrome c-GFP (C) or predominantly mitochondrial localizations (M). (F-I) An α9-helix mutant, P168A mCherry-BAX was not recruited to the mitochondria in the presence of STS and cytochrome c-GFP remained localized at the mitochondria. The appearance of BAX aggregates in these cells does not correspond to mitochondria, and may represent lysosomal uptake of excessive amounts of the fusion protein. (J) A pie chart of scored cells. (K-N) The BAX α5 mutant was also not recruited in the presence of STS, however cytochrome c-GFP was cytosolic in this condition. (O) A pie chart of scored cells. The distribution of cytochrome c-GFP was significantly different in cells expressing the P168A mutant of BAX under these conditions (χ^2^ test, p < 0.0005), while cells expressing WT BAX were not significantly different from cells expressing the α5 mutant protein (p = 0.277). Size bar = 5 μm.
(TIF)

S4 Fig Recruitment of BAX α9 mutant was restored in the presence of wild type BAX in HCT116^*BAX-/-/BAK-/-*^ cells. (A-D) Co-expression of the BAX α9 mutant (P168A mCherry-BAX) and wild type (WT) GFP-BAX in the presence of STS restored the ability of BAX α9 mutant to participate in recruitment to the MOM. A merged image (A) is followed by images of each separate channel. (E) A pie chart of cells scored with predominantly cytosolic BAX (C) or predominantly mitochondrial BAX (M). (F-I) Additional mutations in the α5 region created a double mutant, BAX α5/α9. (J) A pie chart of scored cells. When co-expressed with wild type GFP-BAX, the BAX α5/α9 double mutant failed to participate in BAX recruitment to the MOM (χ^2^ test, p < 0.0005). Size bar = 5 μm.
(TIF)

S5 Fig Recruitment of BAX α9 mutant occurs after wild type BAX recruitment. Time-lapse imaging of a D407 cell co-transfected with wild type GFP-BAX and the BAX α9 mutant (P168A mCherry-BAX) was induced for apoptosis using 1 μM staurosporine (STS). (A-C) Stills from the time-lapse video are shown before wild type BAX recruitment at 120 minutes after STS addition. Both (B) wild type BAX and (C) P168A mCherry-BAX are diffusely distributed. (D-F) Stills from the time-lapse video shown at 139 minutes after STS addition depict (E) wild type BAX recruitment, but (F) diffusely localized P168A mCherry-BAX. (G-I) At 225 minutes after STS addition, both (H) wild type BAX and (I) P168A mCherry-BAX show a punctate pattern indicative of BAX recruitment to the mitochondria. (J) Four regions of interest were identified within the cell, and fluorescence intensity was quantified. The increase in relative fluorescence from the baseline (normalized to one) demonstrates the BAX recruitment process to the mitochondrial membrane over time. The fluorescence for GFP-BAX has reached a plateau at the time when the BAX α9 mutant begins to show an increase in fluorescence. The shaded region depicts the standard deviation among regions of interest.
(TIF)

S1 Video mCherry-BAX recruitment event in a D407 cell. A D407 cell expressing mCherry-BAX and mito-BFP was challenged with 1 μM staurosporine and time-lapse imaging was performed. mCherry-BAX moves from cytosolic to mitochondrial localization. The movie is a series of z-stacked images where each frame represents one minute, and the movie is formatted at 7 frames per second.
(MOV)

S2 Video Synchronous BAX initiation and recruitment in three cell types. GFP-BAX recruitment was monitored after apoptotic induction in a (A) D407 cell (induced by staurosporine (STS)), (B) a HeLa cell (STS), and (C) a HCT116 cell (STS). The movie is a series of z-stacked images captured where one frame represents one minute and each movie is formatted at 7 frames per second. Movies were cropped to align the initiation of BAX recruitment for each cell type. To allow for best visualization, cells are not similarly scaled.
(MP4)

S3 Video mCherry-BAX recruitment concurrent with cytochrome c-GFP release. Cytochrome c-GFP release occurred rapidly relative to mCherry-BAX recruitment. (A) mCherry-BAX and cytochrome c-GFP were monitored after apoptotic induction (1 μM staurosporine) in a D407 cell. (B) Each channel was separated for better representation of the initiation of BAX recruitment coinciding with cytochrome c-GFP release. The movie is a series of z-stacked images captured where one frame represents one minute and the movie is formatted at 7 frames per second.
(MP4)
