# Supplementary figures and images for: Live-cell imaging to measure BAX recruitment kinetics to mitochondria during apoptosis
Source: PLoS One. 2017 Sep 7;12(9):e0184434. doi: 10.1371/journal.pone.0184434 (PMC5589231; doi:10.1371/journal.pone.0184434)

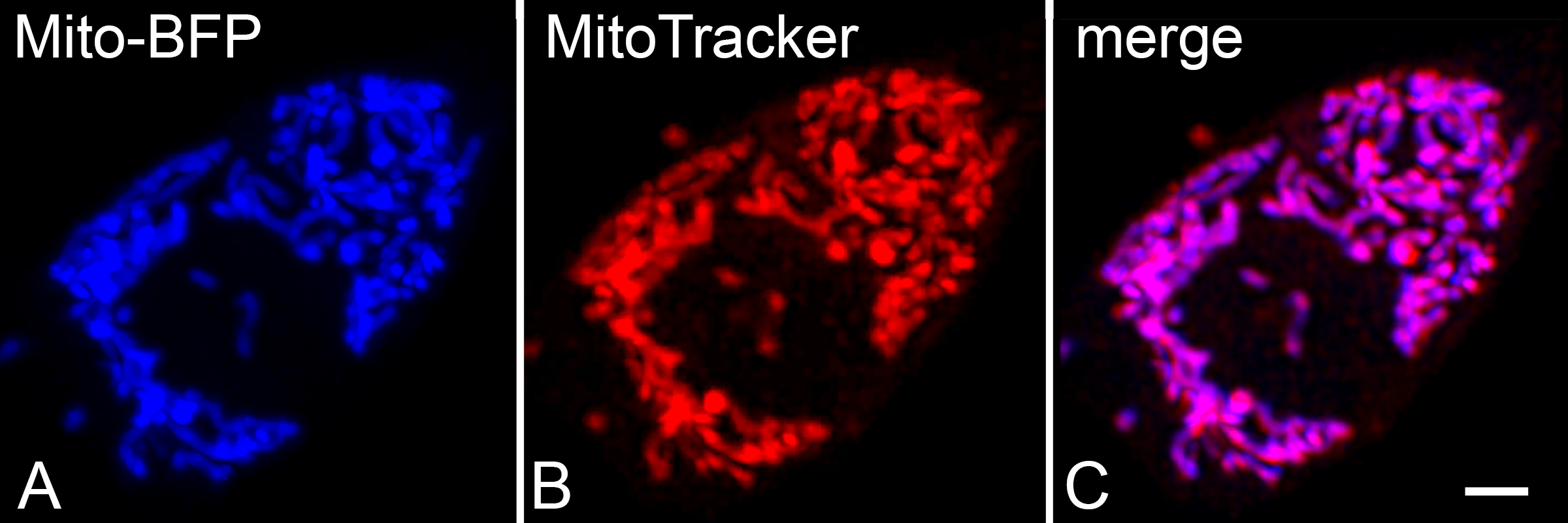

Supplement: S1 Fig — Mito-BFP encodes a transcript containing the mitochondrial targeting sequence for cytochrome c oxidase subunit VIII fused in frame to BFP. Mitotracker Red FM is a live-cell imaging dye that stains the mitochondria. A representative image of a D407 cell expressing (A) Mito-BFP and also stained with (B) Mitotracker Red, shows colocalization of the two markers in (C) a merged image. Size bar = 3 μm. (TIF) [file pone.0184434.s001.tif]

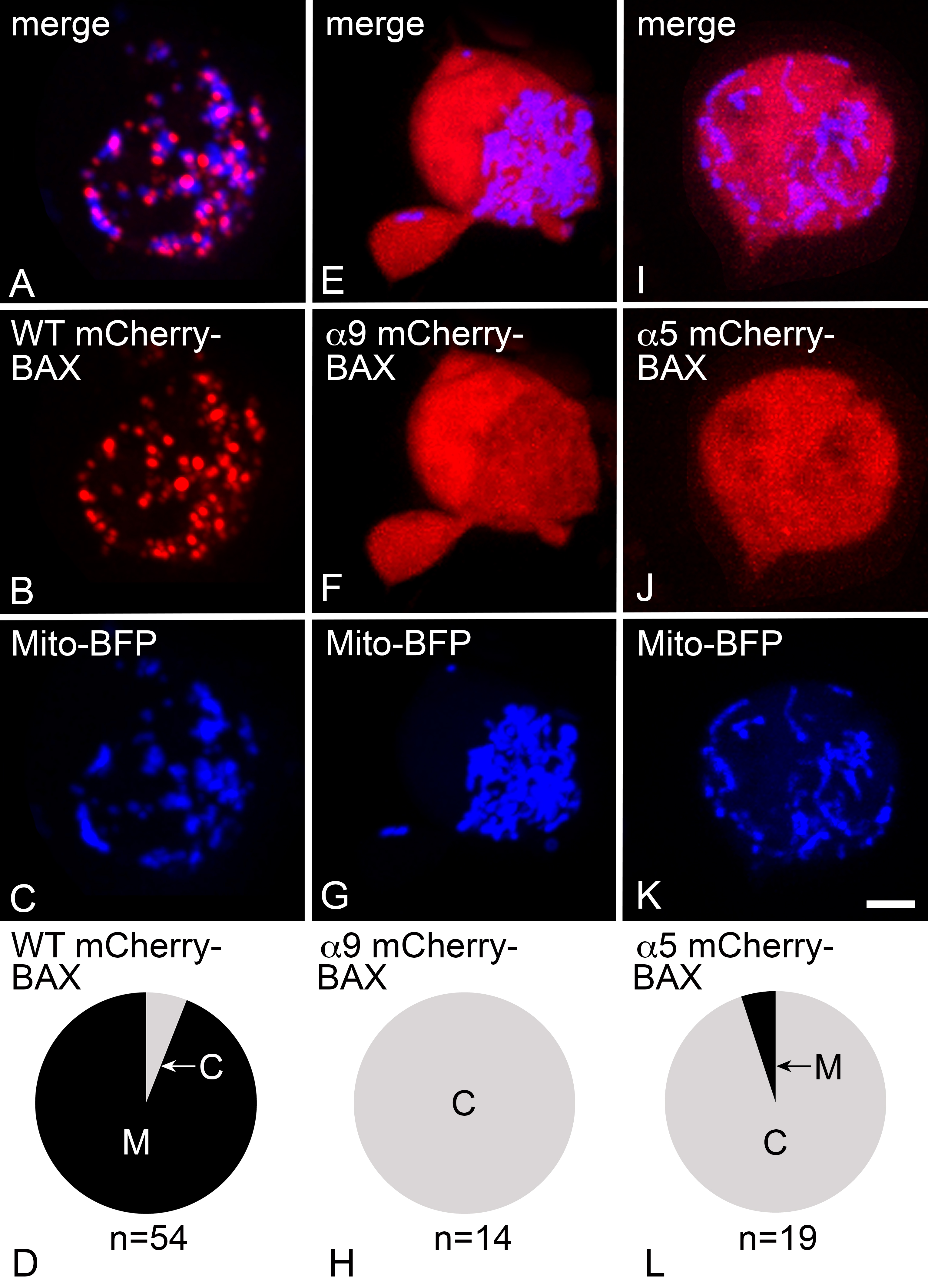

Supplement: S2 Fig — HCT116BAX-/-/BAK-/- cells were nucleofected with different BAX mutants to provide evidence for functioning BAX fusion proteins. Images were taken 18 hours post addition of 1 μM staurosporine (STS), at a time when a majority of these cells exhibit BAX activation, and presented as three panels, a merged image along with individual channels. (A-C) Wild type (WT) mCherry-BAX showed recruitment to the MOM indicated by the punctate pattern co-localized with the mitochondria. (D) A pie chart showing the distribution of the cells showing either predominantly cytosolic (C) or predominantly mitochondrial (M) localization. (E-G) The BAX α9 mutant failed to fully recruit to the MOM, indicated by diffuse localization of BAX α9. (H) Pie chart of scored cells. (I-K) The BAX α5 mutant also failed to recruit to the MOM, indicated by diffuse localization of BAX α5. (L) Pie chart of scored cells. Both mutants show a significantly different localization pattern of BAX compared to the WT protein under these conditions (χ2 test, p < 0.0005). Size bar = 5 μm. (TIF) [file pone.0184434.s002.tif]

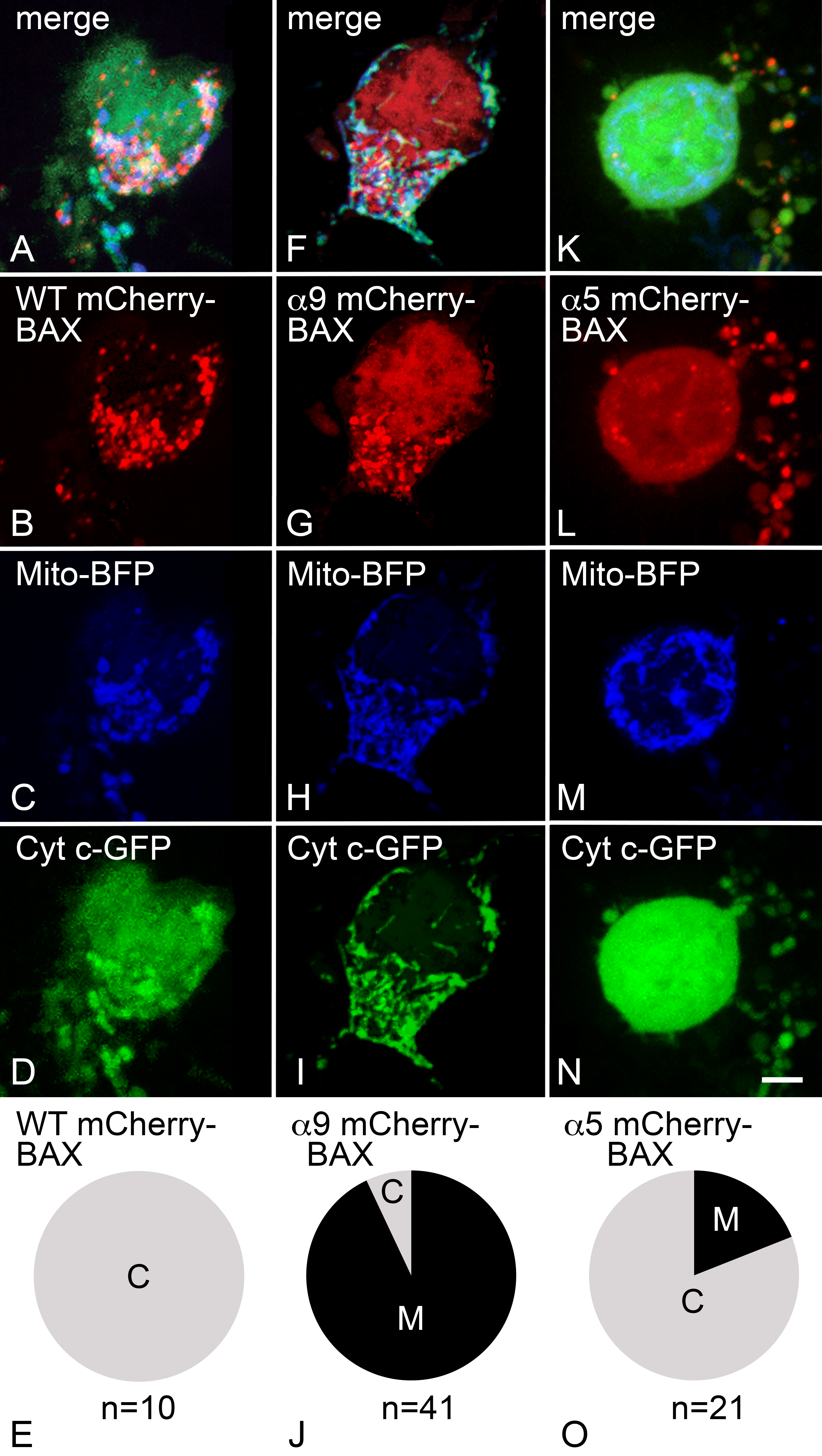

Supplement: S3 Fig — HCT116BAX-/-/BAK-/- cells expressing wild type or mutant mCherry-BAX, cytochrome c-GFP and mito-BFP were challenged with 1μM staurosporine (STS) and observed at 18 hours after treatment. In healthy cells, the cytochrome c fusion protein is localized to mitochondria (see Fig 6 and S3 Video). (A-D) Wild type mCherry-BAX exhibits punctate BAX and diffuse cytochrome c-GFP labeling. The merged image (A) is followed by separate channels. (E) A pie chart showing the scoring of cells exhibiting predominantly cytosolic distribution of cytochrome c-GFP (C) or predominantly mitochondrial localizations (M). (F-I) An α9-helix mutant, P168A mCherry-BAX was not recruited to the mitochondria in the presence of STS and cytochrome c-GFP remained localized at the mitochondria. The appearance of BAX aggregates in these cells does not correspond to mitochondria, and may represent lysosomal uptake of excessive amounts of the fusion protein. (J) A pie chart of scored cells. (K-N) The BAX α5 mutant was also not recruited in the presence of STS, however cytochrome c-GFP was cytosolic in this condition. (O) A pie chart of scored cells. The distribution of cytochrome c-GFP was significantly different in cells expressing the P168A mutant of BAX under these conditions (χ2 test, p < 0.0005), while cells expressing WT BAX were not significantly different from cells expressing the α5 mutant protein (p = 0.277). Size bar = 5 μm. (TIF) [file pone.0184434.s003.tif]

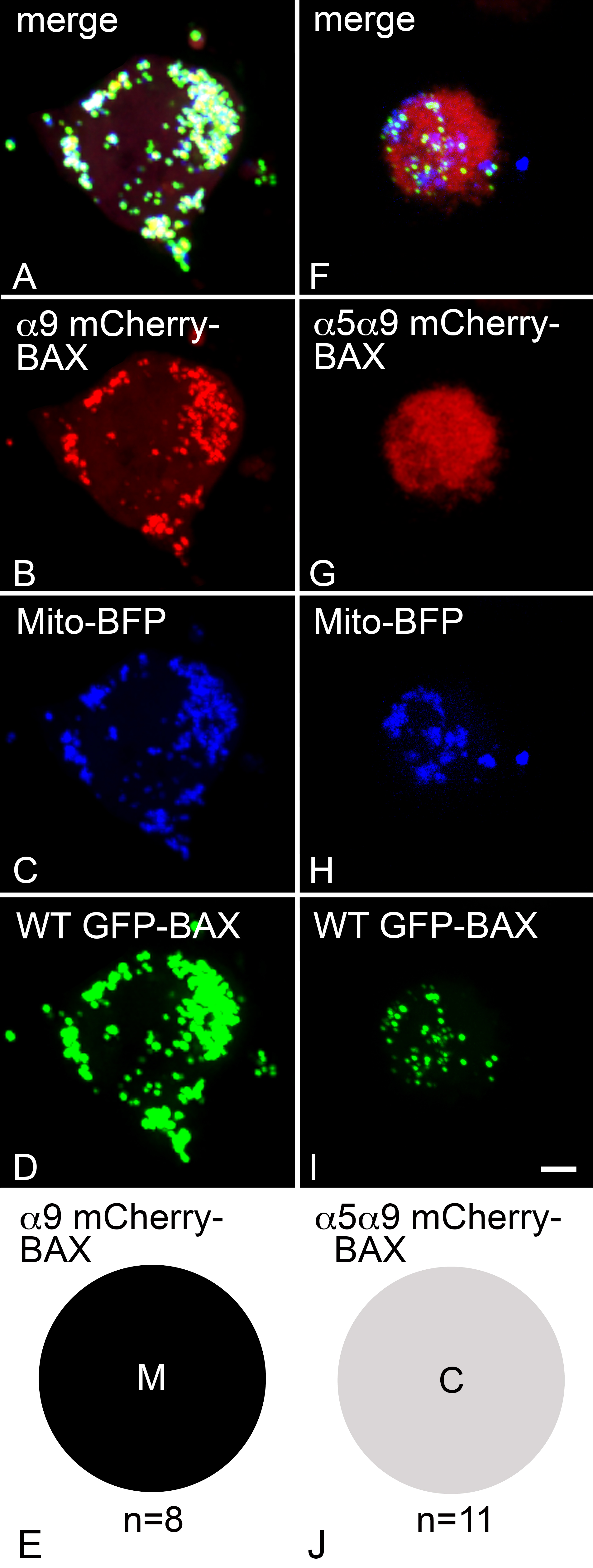

Supplement: S4 Fig — (A-D) Co-expression of the BAX α9 mutant (P168A mCherry-BAX) and wild type (WT) GFP-BAX in the presence of STS restored the ability of BAX α9 mutant to participate in recruitment to the MOM. A merged image (A) is followed by images of each separate channel. (E) A pie chart of cells scored with predominantly cytosolic BAX (C) or predominantly mitochondrial BAX (M). (F-I) Additional mutations in the α5 region created a double mutant, BAX α5/α9. (J) A pie chart of scored cells. When co-expressed with wild type GFP-BAX, the BAX α5/α9 double mutant failed to participate in BAX recruitment to the MOM (χ2 test, p < 0.0005). Size bar = 5 μm. (TIF) [file pone.0184434.s004.tif]

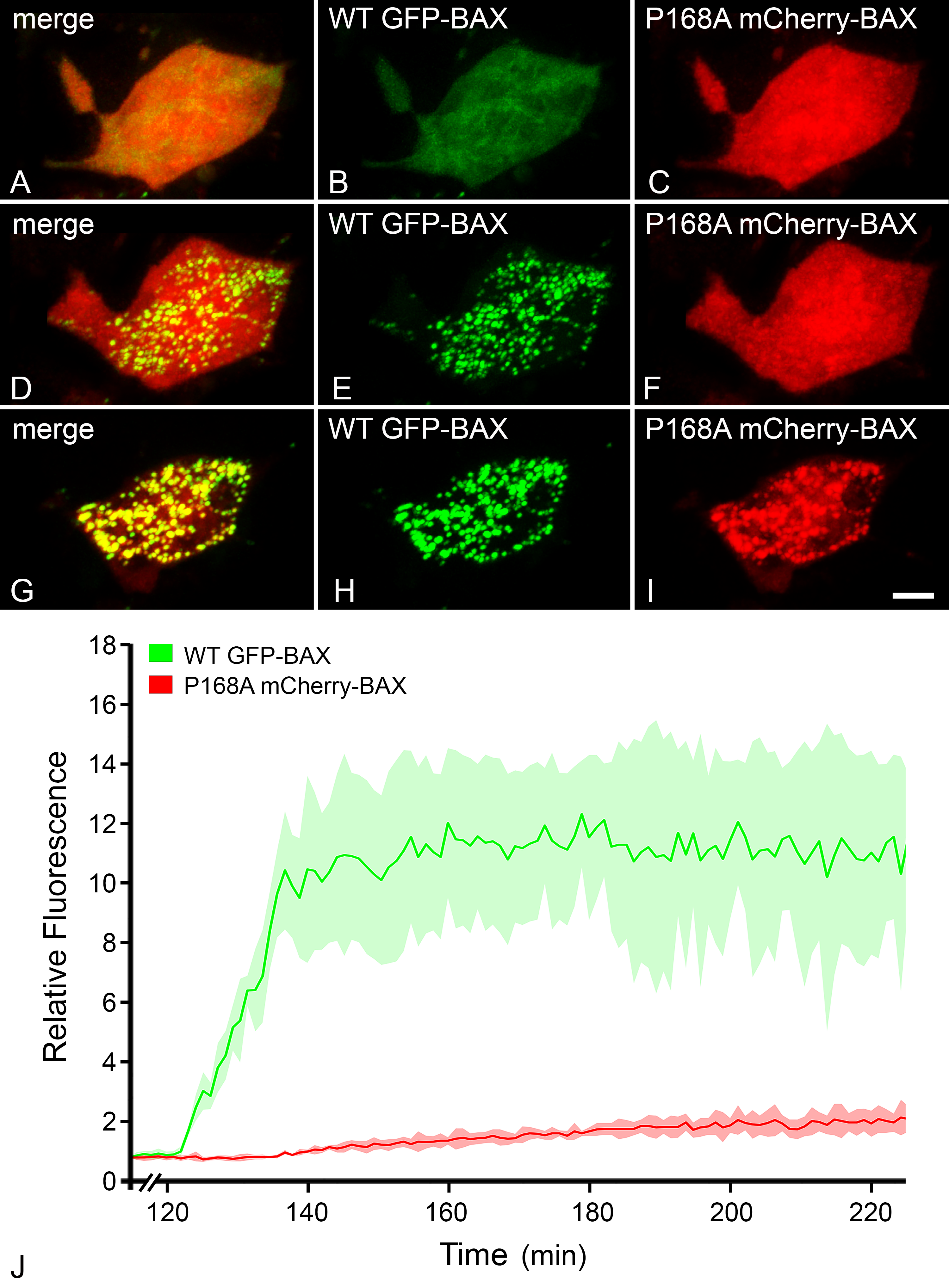

Supplement: S5 Fig — Time-lapse imaging of a D407 cell co-transfected with wild type GFP-BAX and the BAX α9 mutant (P168A mCherry-BAX) was induced for apoptosis using 1 μM staurosporine (STS). (A-C) Stills from the time-lapse video are shown before wild type BAX recruitment at 120 minutes after STS addition. Both (B) wild type BAX and (C) P168A mCherry-BAX are diffusely distributed. (D-F) Stills from the time-lapse video shown at 139 minutes after STS addition depict (E) wild type BAX recruitment, but (F) diffusely localized P168A mCherry-BAX. (G-I) At 225 minutes after STS addition, both (H) wild type BAX and (I) P168A mCherry-BAX show a punctate pattern indicative of BAX recruitment to the mitochondria. (J) Four regions of interest were identified within the cell, and fluorescence intensity was quantified. The increase in relative fluorescence from the baseline (normalized to one) demonstrates the BAX recruitment process to the mitochondrial membrane over time. The fluorescence for GFP-BAX has reached a plateau at the time when the BAX α9 mutant begins to show an increase in fluorescence. The shaded region depicts the standard deviation among regions of interest. (TIF) [file pone.0184434.s005.tif]
